# Case report: Whole genome sequence of *Clostridium perfringens* JUM001 causing acute emphysematous cholecystitis

**DOI:** 10.3389/fmicb.2022.1066880

**Published:** 2022-11-17

**Authors:** Mari Tohya, Tomohiro Otsuka, Jiro Yoshimoto, Yoichi Ishizaki, Teruo Kirikae, Shin Watanabe

**Affiliations:** ^1^Department of Microbiology, Juntendo University School of Medicine, Tokyo, Japan; ^2^Department of Microbiome Research, Juntendo University School of Medicine, Tokyo, Japan; ^3^Department of Digestive and General Surgery, Juntendo University Urayasu Hospital, Chiba, Japan

**Keywords:** *Clostridium perfringens*, emphysematous cholecystitis, whole-genome sequencing, toxinotype A, α-toxin, pCP13-like family plasmid, pCW3-like family plasmid

## Abstract

A strain of *Clostridium perfringens* was isolated from the bile sample of a patient with emphysematous cholecystitis who underwent a laparoscopic cholecystectomy, followed by treatment with meropenem and recovery. Metagenomic analysis of the bile sample showed that 99.73% of the bile microbiota consisted of *C. perfringens*, indicating that *C. perfringens* JUM001 was the causative pathogen of acute emphysematous cholecystitis in this patient. Complete genome sequencing showed that *C. perfringens* JUM001 contained a circular chromosome of 3,231,023 bp and two circular plasmids, pJUM001-1 of 49,289 bp and pJUM001-2 of 47,855 bp. JUM001 was found to possess a typing toxin gene, *plc*, but no other typing toxin genes, indicating that its toxinotype is type A. The plasmids pJUM001-1 and pJUM001-2 belonged to the pCP13-like and pCW3-like families of plasmids, respectively, which are characteristic conjugative and archetypical plasmids of *C. perfringens.* Phylogenetic analysis showed that JUM001 was closely related to *C. perfringens* strain JXNC-DD isolated from a dog in China. To our knowledge, this is the first report of whole-genome sequences of a clinical isolate of *C. perfringens* causing acute emphysematous cholecystitis.

## Introduction

*Clostridium perfringens* is a Gram-positive, obligate anaerobic, spore-forming rod, belonging to the phylum *Bacillota* that has been isolated from various environments, such as soil and sewage, as well as from the intestinal flora of humans and animals (Wells and Wilkins, 1996). *C. perfringens* has been found to cause food poisoning, acute peritonitis and necrotizing enteritis (Wells and Wilkins, 1996). *C. perfringens* produces four major toxins, α, β, ε, and ι; and its pathogenesis and specific symptoms depend on its toxinotype, characterized by sets of produced toxins, including toxins A, B, C, D, E, F, and G (Rood et al., 2018).

Approximately 200,000 patients are diagnosed with acute cholecystitis annually in the US (Gallaher and Charles, 2022). Of acute cholecystitis, emphysematous cholecystitis is a fulminant variant of acute cholecystitis, occurring in about 1% of patients with acute cholecystitis, and having a relatively high mortality rate of 15–25% (Kowalski et al., 2022). Although few studies have evaluated the causative agents of emphysematous cholecystitis, due to the limited number of patients with this condition, *Clostridium* species, *Klebsiella* species, and *Escherichia coli* have been isolated from bile samples of patients with emphysematous cholecystitis (Kowalski et al., 2022).

To date, the complete genome sequences of 52 strains of *C. perfringens* have been deposited in the NCBI database. Of these 52 strains, 21 were derived from human fecal samples, 12 from animal samples, 10 from food samples, six from environmental sources (sludge, soil, and aquarium water) and three from unknown samples. To our knowledge, however, the complete genome sequences of *C. perfringens* causing cholecystitis, including emphysematous cholecystitis, have not been determined. The present study describes the whole-genome sequence of an isolate of *C. perfringens* obtained from a patient with emphysematous cholecystitis who was successfully treated by laparoscopic surgery.

## Case report

**FIGURE 1:**
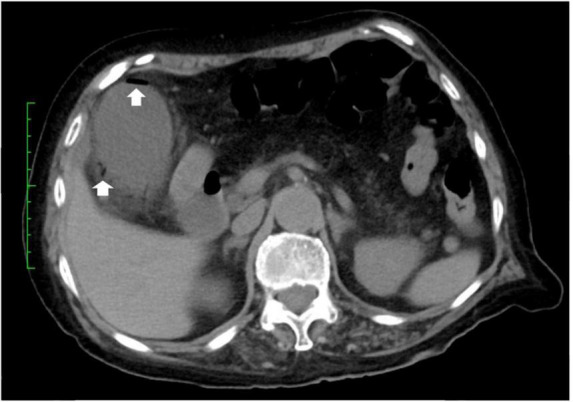
CT-scan of the abdomen. Abdominal CT showing air in the walls (arrows) of the gallbladder.

A 79-year-old woman experienced right upper abdominal pain, accompanied by a fever of 39°C, in August 2021. The following morning, she visited the emergency department at Juntendo University Urayasu Hospital. Blood tests showed a red blood cell count of 2.88 million/mm^3^, a hemoglobin concentration of 10.7 g/dl, a hematocrit of 31.4%, a white blood cell (WBC) count of 7,700/mm^3^, a platelet count of 169,000/mm^3^, and a C-reactive protein (CRP) concentration of 58 mg/l. Abdominal computed tomography (CT) showed an enlarged gallbladder and emphysema in the gallbladder wall (Figure 1). She was diagnosed with emphysematous cholecystitis and underwent a laparoscopic cholecystectomy on the same day. Pathological findings of the surgically removed gallbladder showed acute cholecystitis with serositis. Blood tests on postoperative day (POD) 1 showed a WBC count of 7,700/mm^3^ and an increased CRP level of 174 mg/l. Her abdominal pain was improved. She was treated with meropenem 1 g q8h for 7 days beginning on POD 0. Blood tests on POD 2 showed a WBC count of 5,800/mm^3^ and an increased CRP level of 199 mg/l, and blood tests on POD 5 showed a WBC count of 4,400/mm^3^ and a slightly increased CRP level of 40 mg/l. The patient was discharged from the hospital on POD 8. Her medical history included radical surgery for early-stage lung cancer at age 76 years in July 2018; and craniotomy for necrotic tissue resection of a brain abscess at age 78 years in January 2021, although no causative bacterial agent was identified. She had no history of diabetes mellitus.

## Materials and methods

### Bile sample collection

During cholecystectomy, a bile sample was obtained from the fundus of the gallbladder using an 18-G needle and 20 ml syringe under sterile conditions. An aliquot of the sample was transported anaerobically to the clinical laboratory, and the remaining sample was immediately frozen at −80°C for metagenome analysis.

### DNA extraction and 16S rRNA gene sequencing

DNA was extracted from the bile sample using DNeasy PowerSoil Kits (QIAGEN, Hilden, Germany). A DNA library was prepared in accordance with the *Illumina 16S Metagenomic Sequencing Library Preparation Guide* targeting the V3–V4 region of the 16S rRNA gene. The fastq files were analyzed using QIIME 2 software (Bolyen et al., 2019).

### Bacterial isolation and identification

The bile sample was inoculated onto gifu anaerobic medium (GAM) agar (Nissui Pharmaceutical Co., Tokyo, Japan) and cultured in an anaerobic chamber at 37°C for 24 h. A strain, JUM001, was isolated from the agar and its bacterial species was identified using MALDI Biotyper (Bruker, Billerica, MA, USA).

### DNA extraction and whole-genome sequencing of an isolate

DNA from the isolate, JUM001, was extracted using a DNeasy Blood and Tissue kit (QIAGEN) for MiSeq (Illumina, San Diego, CA, USA) and using a Genomic-tip 20/G kit (QIAGEN) for MinION (Oxford Nanopore Technologies, Oxford, UK). The whole-genome sequences of JUM001 were determined with the MiSeq and MinION sequencers, according to the manufacturers’ instructions. The sequence reads generated by MiSeq were quality trimmed and filtered using CLC Genomics Workbench v11 (CLC bio, Aarthus, Denmark), and the MinION data were base called and adapter trimmed by Guppy v3.6.1 (Oxford Nanopore Technologies). The sequences determined by Miseq and MinION were assembled using Unicycler v0.4.6 (Wick et al., 2017), and the assembled sequences were annotated using Prokka (Seemann, 2014).

### Detection of drug-resistance and toxin genes and comparative genome analysis of the chromosomal sequence of JUM001

The drug resistance genes in the genome sequence of JUM001 were detected using ABRicate v1.0.1,^1^ and the 23 toxin genes listed in Supplementary Table 1 were determined using CLC Genomics Workbench v11. Toxin cluster genes and intergenic sequences of JUM001 were compared with those of other *C. perfringens* strains using BLASTn (Altschul et al., 1990).

### Phylogenetic analysis

Phylogenetic analysis was performed using kSNP3 v3.1 (Gardner et al., 2015) software with a k-mer length of 31. Maximum-likelihood phylogenetic trees were estimated based on the core genome single nucleotide polymorphisms (SNPs) among contigs of JUM001 and other *C. perfringens* strains registered in the NCBI database. Trees were visualized using FigTree v1.4.3.^2^

### Comparative genome analysis of the plasmids, pJUM001-1, and pJUM001-2

The nucleotide sequences of the plasmids pJUM001-1 and pJUM001-2 were compared with sequences of similar plasmids using BLAST. The genetic comparisons of plasmids and their genome structures were visualized by Easyfig (Sullivan et al., 2011).

## Results

Metagenomic analysis of a bile sample obtained from the patient showed that the bile microbiota consisted mainly of *Firmicutes* (99.82%) at the phylum level, *Clostridium* (99.74%) at the genus level and *C. perfringens* (99.73%) at the species level. These findings indicate that *C. perfringens* was the causative pathogen of acute emphysematous cholecystitis in this patient.

**FIGURE 2:**
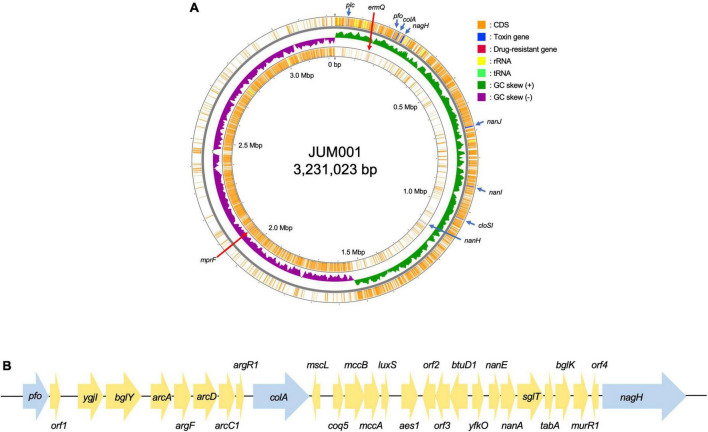
Genetic structure of the JUM001 chromosome. **(A)** Circular structure of the JUM001 chromosome. The figure was generated using the Proksee online tool (https://proksee.ca/). The circular tracks indicate the genes annotated by Prokka on the forward strand (outer orange) and the reverse strand (inner orange) and by GC-skew (green and purple). Toxin encoding genes are indicated with blue arrows and antimicrobial resistance genes by red arrows. **(B)** Genomic environments of three toxin genes, *pfo*, *cola*, and *nagH*, in the JUM001 chromosome. The genes are represented as arrows indicating their transcription orientations and relative lengths. Toxin genes are indicated with blue arrows. *orf1*, gene encoding a septicolysin; *orf2*, gene encoding an ABC transporter permease; *orf3*, gene encoding an ABC-2 family transporter protein; *orf4*, gene encoding a hypothetical protein.

**FIGURE 3:**
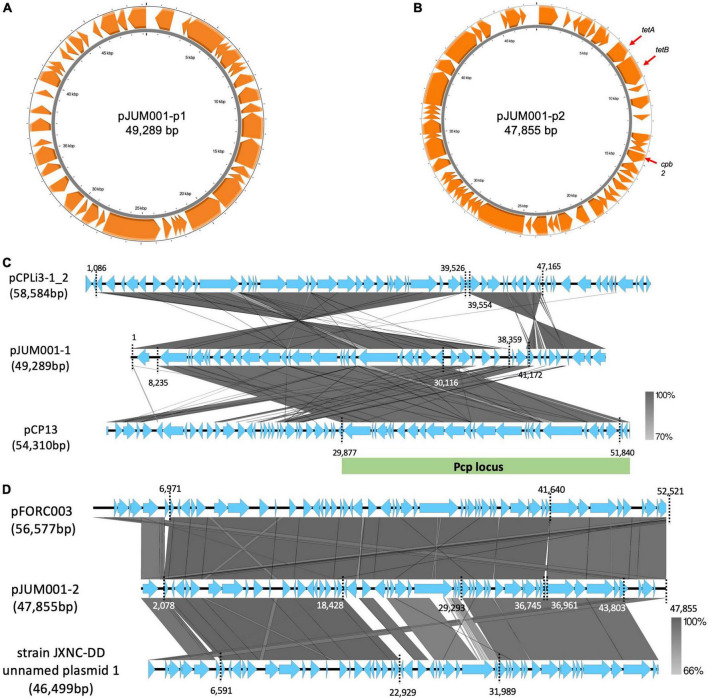
Genetic structures of the two plasmids, pJUM001-1 and pJUM001-2. **(A)** Circular structure of pJUM001-1. The figure was generated using the Proksee online tool. Genes are indicated in orange. **(B)** Circular structure of pJUM001-2. The figure was generated using the Proksee online tool. Genes are indicated in orange. **(C)** Comparison of the plasmid pJUM001-1 with the plasmids pCPLI3-1_2 and pCP13. Homologous regions are shaded in gray, with the percentage identity shaded according to the bar. The Pcp locus is indicated as green bar. **(D)** Comparison of the plasmid pJUM001-2 with the plasmids pFORC003 and JXNC unnamed plasmid 1. Homologous regions are shaded in gray, with the percentage identity shaded according to the bar.

The strain JUM001 was isolated from the bile sample on GAM agar and the bacterial species was identified as *C. perfringens* by MALDI Biotyper. The complete genome sequences of *C. perfringens* JUM001 revealed that this strain consisted of a circular chromosome of 3,231,023 bp, with GC contents of 28.50% (Figure 2A), and two circular plasmids, pJUM001-1 of 49,289 bp (Figure 3A) and pJUM001-2 of 47,855 bp (Figure 3B). The chromosomal sequences had a total of 2,981 CDS, including 96 tRNAs, 37 rRNAs, and 1 tmRNA.

The chromosome of JUM001 was found to contain two drug-resistance genes, *ermQ* (nt 155,935-156,708) and *mprF* (nt 2,066,238-2,067,947), and eight toxin genes, *plc* (nt 48,228-49,424), *pfo* (nt 226,164-227,666), *colA* (nt 239,642-242,956), *nagH* (nt 260,118-265,004), *nanJ* (nt 260,118-265,004), *nanI* (nt 906,579-908,663), *cloSI* (nt 1,032,954-1,034,522), and *nanH* (nt 1,123,343-1,124,491) (Figure 2A). The plasmid, pJUM001-1 did not contain any drug-resistance or toxin genes (Figure 3A), whereas the plasmid pJUM001-2 contained two drug-resistance genes, *tetA* (nt 6,152-7,414) and *tetB* (nt 7,398-9,356), and one toxin gene, *cpb2* (nt 14289-15086) (Figure 3B).

The seven toxinotypes (A–G) of *C. perfringens* strains have been categorized according to the combination of six typing toxins (encoded by toxin genes), including α-toxin (*plc*), β-toxin (*cpb*), ε-toxin (*etx*), ι-toxin (*iap/ibp*), enterotoxin (*cpe*), and NetB (*netB*) (Kiu and Hall, 2018; Rood et al., 2018). In addition, 16 non-typing toxins have been detected in *C. perfringens* (Kiu and Hall, 2018). JUM001 contained only one typing toxin gene, *plc*, indicating that its toxinotype is type A, as well as three non-typing toxin genes, *pfo*, *cloA*, and *nagH*, located close to each other within 38,841 bp (nt 225,164-265,004). As shown by the genetic structure surrounding these three genes (Figure 2B), 40 strains registered in the NCBI database had identical or highly similar DNA sequences, with all 40 being strains of *C. perfringens*, indicating that genetic structures are highly conserved in this species.

JUM001 carried two circular plasmids, pJUM001-1 and pJUM001-2, with the latter harboring two drug-resistance genes, *tetA* and *tetB*. The DNA sequences of pJUM001-1 from nt 1 to 38,359 and from nt 41,172 to 49,289 were 99.18% and 92.05% similar to the sequences of the plasmid pCPLi3-1_2 (CP075919) from nt 1,086 to 39,526 and from nt 39,554 to 47,165, respectively, a plasmid carried by *C. perfringes* CPLi3-1 that had been obtained from sludge (Figure 3C). The DNA sequence of pJUM001-1 from 8,235 to 30,116 was 97.87% similar to the sequence of pCP13 (NC_003042) from nt 29,877 to 51,840, which was carried by *C. perfringes* 13 obtained from soil (Figure 3C). The DNA sequences of pJUM001-2 from nt 2,078 to 36,745 and from nt 36,961 to 47,855 were 99.48 and 95.56% similar to the sequences of pFORC3 (CP009558) from nt 6,971 to 41,640 and from nt 41,639 to 52,521, respectively, a plasmid carried by *C. perfringens* FORC3 obtained from aquarium water (Figure 3D). The DNA sequences of pJUM001-2 from nt 2,078 to 18,428 and from nt 29,293 to 43,803 were 99.16 and 95.34% similar to the sequences of strain JXNC-DD unnamed plasmid 1 (CP102298) from nt 6,591 to 22,929 and from nt 31,989 to 46,499, respectively, which was carried by *C. perfringens* JXNC-DD obtained from a dog (Figure 3D).

**FIGURE 4:**
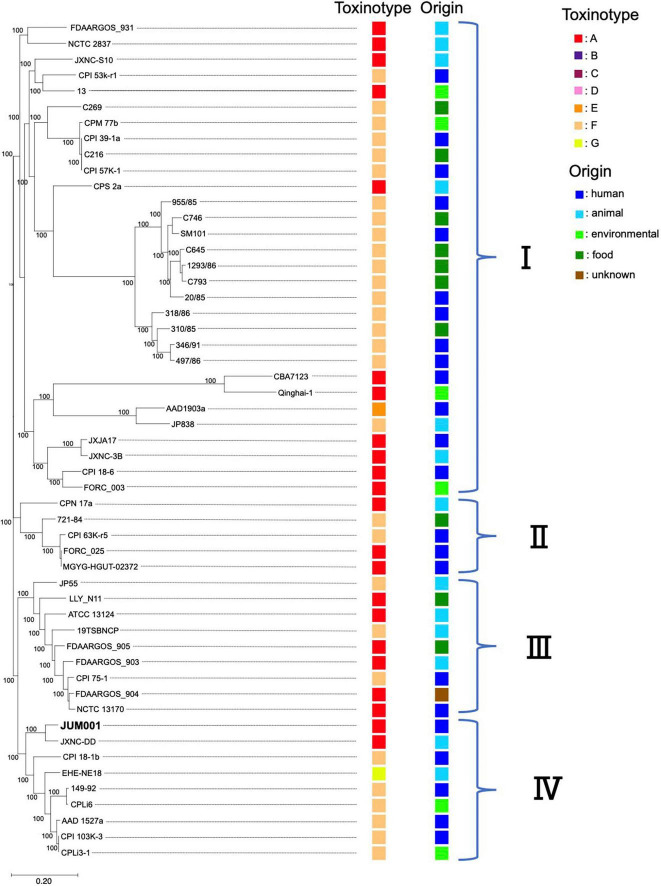
Phylogenetic tree of *C. perfringens* JUM001 and 52 previously described *C. perfringens* strains. The maximum-likelihood phylogenetic using pangenome SNPs among the 53 strains. The toxinotype and origin of isolation of each of these strains are also shown.

Evaluation of the phylogenetic relationships of JUM001 and the 52 *C. perfringens* strains deposited in the NCBI database showed that these *C. perfringens* strains formed four clades (Figure 4). JUM001 was located within clade IV and was most close to strain JXNC-DD, which had been isolated from a dog in China (Figure 4). In addition, pJUM001-2 and plasmids carried by JUM001 and JXNC-DD had highly similar genetic structures (Figure 3D).

## Discussion

*Clostridium perfringens* JUM001, the causative agent of acute emphysematous cholecystitis in this patient, did not have characteristic properties that contribute to specific pathogenesis, as shown by toxin production, phylogenetic relationships and harboring plasmids. First, JUM001 belonged to toxinotype A and produced eight toxins general for this species (Kiu and Hall, 2018). The type A strains are most commonly isolated from environmental sources and associated with food poisoning in humans (Petit et al., 1999). Second, JUM001 belonged to cluster IV in the phylogenetic tree of *C. perfringens* isolates, which were derived from various sources, including humans, animals and environments. Third, the two plasmids carried by JUM001, pJUM001-1, and pJUM001-2, belonged to the pCP13-like and pCW3-like families of plasmids, respectively, both of which are characteristic conjugative and archetypical plasmids of *C. perfringens*.

Plasmids belonging to the pCP13-like family possess a highly conserved Pcp locus (>97% nt identity), which mediates horizontal gene transfer (HGT) by conjugation with a high transfer frequency in *C. perfringens* (Watts et al., 2019). These plasmids have been reported to harbor toxin genes, including a gene encoding a novel binary clostridial enterotoxin (BEC) (Yonogi et al., 2014), although pJUM001-1 did not harbor any known toxins. Plasmids belonging to the pCW3-like family are highly related to conjugative toxin and antibiotic resistance plasmids in *C. perfringens* (Bannam et al., 2006). All pCW3-like family plasmids have been found to carry a tetracycline resistance operon. Moreover, large regions of similarity have been observed between pCW3 plasmids and plasmids that carry the enterotoxin gene, *cpe* (Brynestad et al., 2001; Miyamoto et al., 2006).

Although rapidly progressive infections, such as emphysematous cholecystitis, caused by *C. perfringens* are clinically rare, all causative pathogens should be analyzed bacteriologically and genetically to determine their pathological properties. These analyses should include determinations of their toxinotypes and genotypes, and possibly whole-genome sequencing. Whole-genome sequencing-based analysis of agents causing severe infections, such as emphysematous cholecystitis, is necessary to clarify the pathology of severe infections with *C. perfringens*. To our knowledge, however, no previous study has reported the whole-genome sequences of the causative isolates, although there are three case reports of emphysematous cholecystitis caused by *C. perfringens* (Cochrane et al., 2015; Hashiba et al., 2016; Koole et al., 2016). The first patient was an 82-year-old man who developed a liver abscess with severe hemolytic anemia, with multiplex PCR detecting the *cpa* gene, indicative of toxinotype A (Hashiba et al., 2016). The second patient was a 65-year-old woman with *C. perfringens* septicemia secondary to emphysematous cholecystitis as well as lever abscesses, although the isolate was not described (Cochrane et al., 2015). The third patient was a 46-year-old man with *C. perfringens* cholecystitis with a circular gas pattern in the gallbladder and bile ducts, although this isolate was also not described (Koole et al., 2016).

To our knowledge, this is the first report of whole-genome sequences of a clinical isolate of *C. perfringens* causing acute emphysematous cholecystitis. The isolate belonged to toxinotype A and harbored eight toxin genes, including *plc* encoding α-toxin. However, a limitation of this study is that it included bacterial analysis of a single isolate, therefore it is difficult to conclude the relationship between bacteriological properties and acute emphysematous cholecystitis. Genomic information may extend the pathophysiologic understanding of *C. perfringens* virulence factors.

## Accession numbers

The genome sequence data of a bile sample and an isolate JUM001 have been deposited in the DNA Data Bank of Japan (DDBJ) Sequence Read Archive under accession numbers DRA014765 and DRA014766, respectively. The assembled and annotated whole nucleotide sequences of the chromosome and plasmids have been deposited in the GenBank database under accession numbers AP026870, AP026871, and AP026872, respectively.

## Data availability statement

The datasets presented in this study can be found in online repositories. The names of the repository/repositories and accession number(s) can be found below: https://www.ncbi.nlm.nih.gov/genbank/, AP026870; https://www.ncbi.nlm.nih.gov/genbank/, AP026871; and https://www.ncbi.nlm.nih.gov/genbank/, AP026872.

## Ethics statement

This study was approved by the Ethics Committee of Juntendo University, Urayasu Hospital (approval no. 3-009). Written informed consent was obtained from a patient before surgery for publication of this report.

## Author contributions

SW was responsible for conception and design. MT performed isolation of the bacterial strain and analyzed genomic data. TO, JY, and YI performed the surgery operation. MT and TK wrote the manuscript. All authors read and approved the final manuscript.

## Footnotes

1
https://github.com/tseemann/abricate

2
http://tree.bio.ed.ac.uk/software/figtree/
