# Supplementary material for: Case report: Whole genome sequence of Clostridium perfringens JUM001 causing acute emphysematous cholecystitis
Source: Front Microbiol. 2022 Nov 17;13:1066880. doi: 10.3389/fmicb.2022.1066880 (PMC9714627; doi:10.3389/fmicb.2022.1066880)
Supplement: Supplementary file 1 [file Table_1.pdf]

Supplementary Table 1: The toxin gene profile of C. perfringens JUM001.

	Toxin genes	Toxin	JUM001
Typing toxin	*plc/cpa*	phospholipase	+
	cpb	β-toxin	-
	etx	ε-toxin	-
	iap	ι-toxin compomrmt A	-
	ibp	ι-toxin compomrmt B	-
	cpe	Enterotpxin (CPE)	-
	netB	NetB	-
Non-typing toxins	*cpb2*	β2 toxin	-
	lam	λ-toxin	-
	pfo/pfoA	Perfringolysin O	+
	cpd	δ-toxin	-
	ccp/cloSI	Clostripain	+
	colA	Microbial collagenase	+
	nanl	Sialidase	+
	nanJ	Exo-α-sialidase	+
	nanH	Neuraminidase	+
	nagH	Hyaluronidase	+
	tpeL	Glucosylating toxin	-
	becA	Binary Enterotoxin Component A	-
	becB	Binary Enterotoxin Component B	-
	netE	NetE	-
	netF	NetF	-
	netG	NetG	-
